# Sustainable Valorization of Grape-Leaf-Based Flavonoid Natural Dye Molecules for Eco-Friendly Wool Yarn Dyeing

**DOI:** 10.3390/molecules31101672

**Published:** 2026-05-15

**Authors:** Noman Habib, Fatima Irfan, Tanvir Ahmad, Jamiu Mosebolatan Jabar, Shahid Adeel, Fiaz Hussain, Meral Ozomay, Mansoor Ali

**Affiliations:** 1 Department of Botany, Government College University Faisalabad, Faisalabad 38000, Pakistan; drnomanhabib@gcuf.edu.pk; 2 Department of Applied Chemistry, Government College University Faisalabad, Faisalabad 38000, Pakistan; fatimairfan8128@gmail.com; 3 Department of Statistics, Government College University Faisalabad, Faisalabad 38000, Pakistan; dr_tanvir@gcuf.edu.pk; 4 Textile and Polymer Research Laboratory, Chemistry Department, The Federal University of Technology, P.M.B. 704, Akure 340110, Nigeria; jmjabar@futa.edu.ng; 5 Department of Mechanical Engineering, Gachon University, Seongnam-si 13120, Gyeonggi-do, Republic of Korea; 6 Department of Textile Engineering, Marmara University, Istanbul 34854, Turkey; meral.akkaya@marmara.edu.tr; 7 Department of Chemical Engineering, Sungkyunkwan University, Suwon-si 16419, Gyeonggi-do, Republic of Korea; malimorai14@gmail.com

**Keywords:** Box–Behnken design, flavonoid dye, grape leaves, natural dye molecules, myrobalan, red sumac, wool yarn

## Abstract

The utilization of plant isolates as dyes in applied fields has gained considerable interest due to growing environmental concerns associated with the toxic synthetic dyes. In the present work, the agro-waste (such as grape leaves) has been valorized as a rich source of flavonoid-based natural dye for sustainable dyeing of woolen yarn. Microwave irradiation was further applied to dye molecules and wool yarn to enhance the dye uptake and process efficiency. Processing parameters for dyeing were optimized using Box–Behnken as a statistical design, and the results of the analysis revealed that the processing parameters, including temperature (80 °C), time (25 min), pH 5, and salt concentration (3 g/100 mL), significantly influence the color strength. The microwave irradiation of both flavonoid-based grape leaf extract and yarns up to 4 min, followed by dyeing, has given improved color yield up to a K/S value of 5.38. Metal mordants and bio-mordants were analyzed to improve dye fixation and sustainability. The pretreatment of yarn with Fe^2+^-salt, post-treatment with Al^3+^-salt, and addition of tannic acid during the dyeing process improved the color strength and dye fixation. Furthermore, the addition of red sumac extract during the dyeing of yarns and the pretreatment of yarn with myrobalan as bio-mordants increased the color depth. The colorfastness rating shows that mordanting has improved color stability and has offered maximum resistance to color fading. It is concluded that agro-waste valorization, statistical modeling coupled with radiation treatment, has not only added value in process optimization but also mordanting in the coloring of yarn with grape leaf extract has valorized the green dyeing.

## 1. Introduction

Green products are gaining global attention because their production and applications need fewer toxic auxiliaries. Their existence does not cause pollution, and even their waste can be recycled [1]. Moreover, green products use less energy, solvents, and resources to elute greenhouse gases. Hence, green products have found a strong place among other performance materials for versatile industrial applications such as pharmaceuticals, agriculture, packaging, and textiles [2]. Among green products, natural dyes are particularly important because they are isolated from renewable sources, and mostly, the agricultural wastes are effectively utilized [3]. These dyes are widely used in various potential areas, including food and flavoring, textile dyeing, dye-sensitized solar cells, pharmaceuticals, and Ayurvedic medications [4].

**Figure 1:**
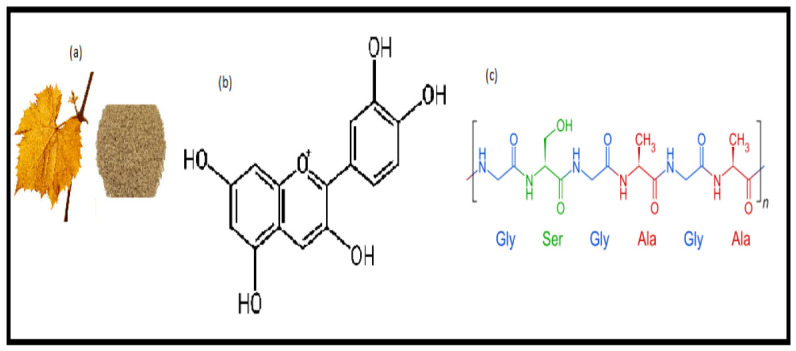
(**a**) Grape leaf powder; (**b**) flavonoid; (**c**) functional unit of wool yarn.

The recent widespread campaign for the use of natural dyes is due to the recent discovery of the toxic nature of the synthetic dyes [5]. Synthetic dyes and intermediates, when released into the environment, can cause serious problems such as cancer, waste pollution, soil depletion, and airborne diseases [6]. As a result, eco-agencies have advocated for a ban on harmful synthetic dyes and encouraged the use of green dyes and the introduction of green dyes in all fields [7]. Even though green dyes offer environmental, medicinal, and economic benefits and are suitable for a wide range of applications, yet low yield and poor color fastness properties limit their industrial applications [8]. Recent solvent-efficient techniques, such as microwave irradiation and statistical optimization tools like Box–Behnken design (BBD), have been explored to improve dyeability and process efficiency. Microwave rays cause rapid mass transfer into the solvent and upscale the yarn surface by peeling the fiber to enhance its uptake ability. Using the Box–Behnken design (BBD) design, not only are dyeing variables selected, but also their responses, such as color depth (K/S), are optimized. This design also helps in reducing carbon, water, energy, cost, and labor [9]. However, their integrated application for specific agro-waste is still limited. To overcome demerits like poor fastness, mordants such as electrolytes of metals and biomolecules are used [10]. However, owing to toxicity, only the salts of Al and Fe or organic acid (TA, tannic acid) are used as chemical additives [11]. Biomolecules are plant phenolics; usually, tannin-based sources are now being used in terms of making the dyeing process sustainable, green, and viable for the globe [12]. There are various sources of plant-based green dyes; however, one of the waste agro-based sources is grape leaves (Figure 1a) [13].

In leaves, various phytochemicals are present, but flavonoid, a polyphenol (Figure 1b), is responsible for imparting a yellow–beige color onto wool. However, various tints are obtainable from natural sources, depending upon the nature of the textiles (fabric or yarns), dyeing methods, and mordants used [14]. The unique chemical structure of polyphenols obtained from grape leaves agro-waste make is suitable for versatile industrial applications such as medical, cosmetics, food, and green synthesis of multifunctional nanoparticles and sustainable textile dyeing [15,16]. Although grape leaf agro-waste has been identified as a promising source of flavonoid-based functional dyes with multifunctional potential [14,15,16], there is a lack of comprehensive studies combining microwave-assisted extraction, statistical optimization, and sustainable bio-mordanting approaches for efficient and eco-friendly dyeing of wool substrates. So, there is a dire need to develop an integrated, sustainable, and optimized dyeing technique using the grape leave agro-waste-based dyes with improved performance and suitability for industrial applications.

In this study, grape leaves extract has been used as a natural dye for the sustainable dyeing of wool yarn. Wool, being a natural protein fiber, has keratin as its main protein unit, where the presence of amido linkage (–C=O, –NH) helps in binding with dye molecules [17]. In view of the Box–Behnken design for product development, MW treatment for isolation yield and bio-mordants to develop colorfast shades has been designed [18]. The microwave-assisted extraction of dye molecules extracted from the grape leaves (agro-waste) was performed, and sustainable dyeing at the optimized processing parameters, determined by Box–Behnken experimental design, was carried out. The results indicated that wool-dyeing behavior is influenced by the processing parameters, and color depth can be improved using bio-mordants. To the best of the authors’ knowledge, no one has explored the microwave-assisted, facile, and sustainable dyeing behavior of grape leaves based on flavonoid dye for wool yarns using a statistical optimization approach compounded with bio-mordants.

## 2. Materials and Methods

### 2.1. Collection and Preparation of Material

The crude dried grape leaves (GLs) as a source of natural flavonoid colorant were provided by the Department of Textile Engineering, Yazd University, Yazd, Iran. The ground leaves were pulverized into powder form (GLP) and screened through a mesh of 20 μm size. The selected bio-anchors, such as red sumac flowers (*R. typhnia*) and myrobalan fruit (*T. chebula*), were also dried, ground finely, and passed through a screen of 20 μm mesh to obtain equal-sized particles in powdered form. On a commercial scale, the salt of Al^3+^ (Al(CH_3_COO)_3_ = A.A) and Fe^2+^ (FeSO_4_ = I.S) and organic acid, such as tannic acid (T.A.) as green chemical mordant were purchased from a textile market, Faisalabad, in Pakistan. Wool yarns were provided by the Department of Carpet, University of Sistan and Baluchestan, Zahedan, Iran, which were washed with neutral soap at 60 °C for 15 min.

### 2.2. Selection of Dyeing Variables

Extraction of dye from screened grape leaves powder (GLP), 4 g, was boiled with 100 mL of distilled water, and filtered. The filtrate (GLPE) containing flavonoid-rich dye was stored for the dyeing process. For the selection of dyeing variables, a statistical approach has been taken, where the Box–Behnken design as a form of response surface methodology has been adopted (Table 1).

The role of the extract’s pH, dyeing time, dyeing temperature, and salt amount has been considered. Owing to the nature of yarn, the pH was 3, 5, 7, and time was 25, 45, and 65 min, temperature was 60, 70, and 80 °C, whereas for maximum exhaustion of dye bath with leveled shading, the salt amount taken was 1, 3, and 5 g/100 mL to obtain the experimental design for the section of dyeing parameters. A design of 27 experiments was formulated, and each experiment was run systematically as presented in Table 1. The results were analyzed for process optimization and level of dyeing variable using analysis of variance through Minitab (version 17) and Design Expert 13.

### 2.3. Effect of Irradiation Process

To observe the role of microwave radiation (MW), three different strategies were used. In the first series of experiments, the filtrate and yarns were given MW treatment for up to 10 min with a difference of 2 min using a high-power commercial MW source (Dawlance = 700 W, 2450 MHz). The dyeing of wool yarn (1 g) with 25 mL of GLP extract (5 pH) having 3 g/100 mL of table salt, after radiation (DAR), was performed at 80 °C for 25 min. In the second series, the dyeing of wool yarns (1 g) with 25 mL of GLP extract (5 pH) having 3 g/100 mL of table salt was performed; after dyeing (RAD), MW radiation was given up to 10 min with an interval of 2 min. For comparative analysis, the dyeing of wool yarns (1 g) with 25 mL of GLP extract (5 pH) having 3 g/100 mL of table salt was performed under microwave radiation (DUR) up to 10 min with an interval of 2 min.

### 2.4. Physiochemical Analysis of GLP Extract and Wool Yarns

Wool yarns before and after radiation, as well as grape leaf extract, were subjected to identification of functional groups through Fourier-transformed infrared spectroscopy (FTIR), where spectra were recorded within the range of 4000–400 cm^−1^ using ATR-FTIR (PerkinElmer Spectrum IR 10.6.1, Shelton, CT, USA). For observing the physical modification of the yarn’s surface after radiation, the fibers were examined through a scanning electron microscope (SEM = 5.0 KV; 46 mm, Hitachi, Tokyo, Japan), and images were scanned at a magnification power of 1000X.

### 2.5. Process of Enhancing the Shade (Mordanting)

The green mordanting process is the backbone of natural colorants for yarns. Owing to toxicity, in this study, sustainable mordants have been employed. The salt solutions of Al^3+^ (A.A), Fe^2+^ (I.S), and tannic acid (T.A) were prepared by dissolving 2 g of each in 100 mL of water. Then, 25 mL of each of the mordant solutions was used to coat the yarns before, after, and during dyeing at 60 °C for 45 min. Bio-mordanting was also performed before, after, and during dyeing of yarns with GLPE at 60 °C for 45 min. For this purpose, 2 g of fine powder of red sumac and myrobalan were boiled with 100 mL of water. After boiling and filtration, 25 mL of each of the bio-mordants was employed to coat the yarns before, after, and during dyeing.

### 2.6. Colorimetric Analysis of Dyed Yarns

The dyed yarns were assessed using the Kubelka Equation by Colori-Spectrophotometer CS 410 (CHNSpec Technology, Taizhou, China) equipped with D 65 source of light at observation of 10°. The colorfastness properties of yarns mordanted before, after, and during dyeing were checked using standard methods given by ISO. Light fastness of dyed yarns was taken by exposing them under Xenon light as per ISO 105-B02:2014 [19]. For this, a blue wool scale (rating 1–8) was used for the analysis of the results, and higher values correspond to better resistance to fading of the prepared dyed wool samples. Similarly, crocking of yarns (rubbing fastness) was taken in dry and wet conditions by rubbing them as per ISO 105-D02 [20]. For rubbing fastness, a bundle of yarns was arranged in parallel to form a uniform surface, and then the crocking finger of the crockmeter moved in a to-and-fro motion over the yarn bundle using standard turns. The fading to washing was assessed after soaping the dyed yarns with neutral soap under the conditions given in ISO 105-C10 [21]. For washing fastness, the grey scale was used for the evaluation of the results, and the reported grades correspond to the change in color of the developed grape-leaf-extract-based dyed yarns [22].

## 3. Results and Discussion

Dyeing wool with agro-waste extract is a challenging task, because wool has a complex structure, where its amide linkage works under certain pH to bind with –OH of the dye molecules [22]. Hence, the statistical modelling using Box–Behnken design has been used to find the dyeing variable for wool coloration using grape leaf extract (Table 1).

### 3.1. Optimization of Dyeing Parameters

The pH of the dyeing medium plays a critical role in the dyeing efficiency of the wool yarns. It is obvious from the results (Table 1) that optimal dye uptake was achieved when grape leaf extract at pH 5 is used to dye wool yarns for 25 min at 80 °C by adding 3 g/100 mL of table salt as a levelling agent with excellent color yield (K/S = 4.7719). The significance of dyeing variables was analyzed through two-way analysis of variance. It can be seen from Table 2 that the fitted model was highly significant (*p* = 0.003) with a strong linear relationship (*p* = 0.001). The fitted model was highly significant (*p* = 0.003) with a strong linear relationship (*p* = 0.001). Statistically (Table 2), the role of pH as an individual factor is significant at the 2% level (*p* = 0.022), and in the two-way interaction, its role with dyeing time (25 min) is also significant at 1% of the level (*p* = 0.01).

Wool yarns have keratin that contains amino (–NH_2_), hydroxyl (–OH), and carboxyl (–COOH) functional groups, that play a pivotal role in the natural dye adsorption. At pH 5, wool fibers develop an ionic bonding with the dye molecules to give a shade of high strength. Not only ionic bonding but also hydrogen bonding and van der Waals interactions between the –OH functional groups of flavonoid dye molecules and –NH, and –COOH functional groups of wool fiber are also responsible for the dyeing of the resultant fiber. Furthermore, the mordants and electrolytes present in the dye bath also facilitate the dyeing process via a coordination and bridging mechanism. Under too-acidic conditions and too-alkaline conditions, the fibers’ strength is disturbed; here, due to their coloration, the fibers cannot hold the colorant molecules firmly. Thus, an acidic dye bath (pH = 5) helps in ensuring fixation and shade uniformity through electrostatic attraction by improving dyeing efficiency towards plant-based colorants in yarns. Hence, the pH of the dye bath for wool yarns was adjusted up to 5 to obtain a high yield. This observation agreed with the earlier report on the natural dyeing of wool fabric [23].

Time and temperature are both prime variables because they are the contact of dye molecules with the yarn matrix for particular intervals at a specific temperature range. Better penetration of dye molecules into the linear structure of wool yarns is due to heat at a certain level for a certain time [22,24]. At 80 °C for 25 min, levelled and uniform dyeing can be achieved because too long dyeing time can cause desorption, whereas too-high heat can cause the desaturation of the actual colorant, where hydrolytic products might be sorbed and show less depth (Table 2). Statistically, it can be seen that the role of time (25 min) and temperature (80 °C) in wool yarn dyeing has been found highly significant (*p* = 0.00). Similarly, the dyeing of wool yarns at 80 °C in the presence of salt (3 g/100 mL) has also been found to be highly statistically significant (*p* = 0.00). The role of salt in wool dyeing is good because it promotes the rate of dyeing by shifting the dye molecules onto yarns through efficient exhaustion [25]. Basically, the wool fibers, when immersed in water, carry a negative charge, whereas dye, if it is also acidic in nature, can create a negative charge, which causes a repulsion against interaction. Salt added helps to neutralize this situation for firm fixation, thereby creating an interactive atmosphere around dye, solvent, and yarn [26]. On adding 3 g/100 mL of table salt during dyeing of wool yarns at 80 °C for 25 min with grape leaf extract of pH 5, the lightest yield was found. Statistically, its role has been found to be significant at the 1% level (*p* = 0.01).

**Figure 2:**
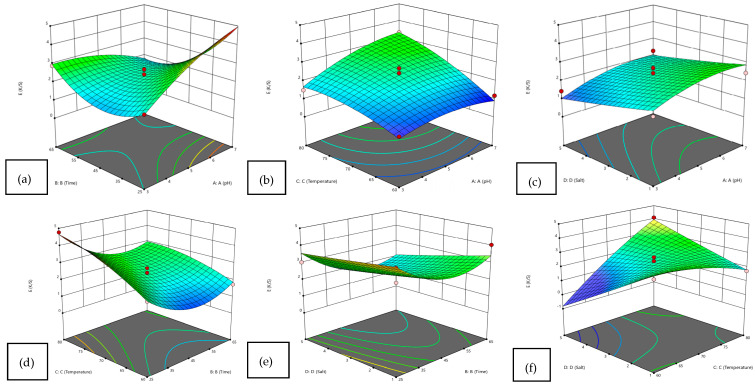
Three-dimensional surface plot for experimental performance analysis: Time*pH (**a**), Temperature*pH (**b**), salt*pH (**c**), Temperature*time (**d**), salt*time (**e**) and salt*temperature (**f**).

Figure 2a–f shows how the response **K/S (%)** varies with **pH** and **time**. The surface indicates a **nonlinear relationship**, where **K/S (%) increases with increasing pH and longer time**, reaching its maximum at high pH and high time values. The plot highlights a **strong interactive effect of pH and time on performance**, helping identify optimal operating conditions. Similarly, Figure 2f shows maximum response observed at **high temperature and low salt levels**, indicating a **strong interactive effect**, with temperature being the dominant factor and salt having an inverse influence. Overall, statistical analysis recommended that dyeing wool yarns at 85 °C for 25 min using grape leaf extract at pH 5 should be performed to obtain the required results. Hence, the low value of standard deviation and R^2^ (91.78%) shows the accuracy of the results obtained in dyeing of wool yarns with grape leaf extract.

**Figure 3:**
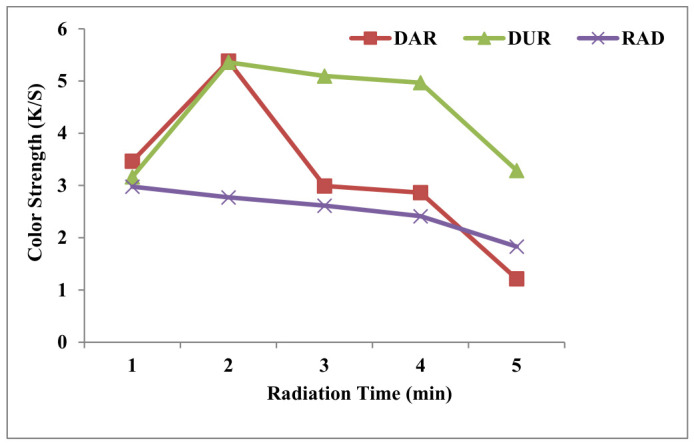
Color strength of wool yarns dyed before (RAD), after (DAR), and under radiation (DUR) at selected conditions (1 = 2 min, 2 = 4 min, 3 = 6 min, 4 = 8 min, and 5 = 10 min).

Microwave radiation always has a promising role in industries due to its rapid and uniform act of treatment. In textiles, for protein fabrics and yarns, heating causes better penetration by surface enrichment to uptake dye up to the maximum extent. Physical treatments, through conduction and connection, take a lot of energy and time to produce desired microwave transfer energy into plant cells through solvent (colorant) molecules and cause efficient mass transfer, which, on coloration, shows high yield [26]. The results portrayed in Figure 3 show that dyeing after radiation (DAR) to both extracts, the yarns have given color depth up to 5.3815. Similarly, dyeing yarns under MW rays (DUR) for 4 min has given a better yield (K/S = 5.360). Comparatively, after dyeing yarns with GLE, the radiation has furnished a shade of good strength (K/S = 2.9793). Thus, the radiation to both GLE and wool yarns, followed by dyeing, has the potential to give excellent results. This improvement also has two-fold benefits. Firstly, the improvement in isolation yield by breaking large clusters of molecules through mass transfer with better penetration gives excellent results. Secondly, the surface scaling of wool yarns is physically in such a way that their substantivity has been enhanced.

### 3.2. Characterization of Wool Yarns

**Figure 4:**
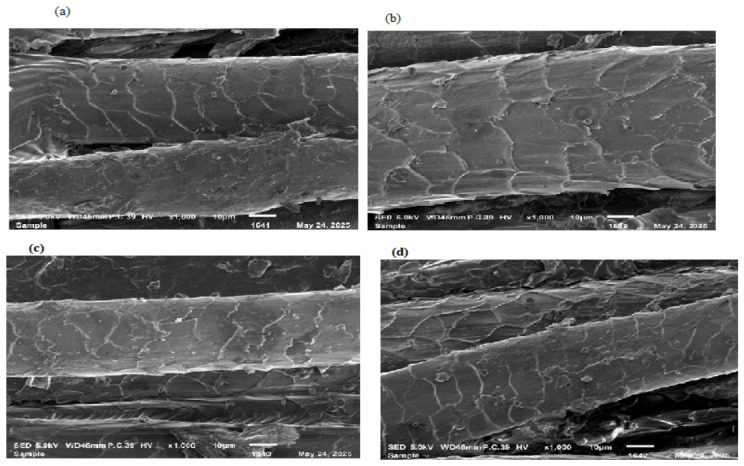
Surface morphology of wool yarns (**a**) before radiation and (**b**) after irradiation; (**c**) dyed wool yarns before radiation and (**d**) after radiation.

**Figure 5:**
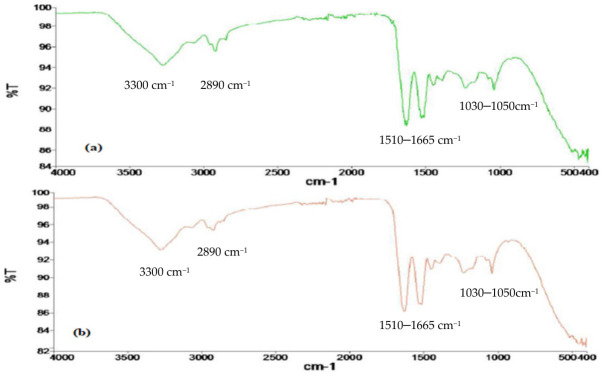
Spectral representation of functional groups in wool yarns (**a**) before and (**b**) after radiation.

The surface morphology of yarns before and after treatment was determined; the results are shown in Figure 4. It can be seen from the results that the surface has been peeled enough to enhance dye uptake of the wool yarns (Figure 4a,b). Also, without treatment, dyed yarns and irradiated dyed yarns on scanning reveal that the fiber has absorbed dye up to the maximum extent (Figure 4c,d). Surface morphological analysis clearly indicates the surface delamination effect. MW causes a rapid and uniform internal heating that results in the localized stress in the fiber surface, leading to compact cuticle layer delamination. This surface modification increases the dye accessibility to wool fiber by developing additional active sites, resulting in enhanced dye diffusion into the fibers. Hence, surface morphology of the specimens confirms the physical modification of yarns, and it is important to note that the SEM and FTIR (Figure 5) results are compatible with each other.

It has also been noted that no chemical changes have been observed in the wool yarns after microwave (MW) radiation through infrared spectral intensities (Figure 5a,b), which revealed that the functional peaks of carbonyl and amino linkages have not been altered before or after treatment. The functional peaks of about 3300 cm^−1^ for –NH group, of about 2890 cm^−1^ for –CH_3_, of about 1510–1665 for –CN/C=O, and of about 1030–1050 cm^−1^ for –S=O reflect that there is no such alteration in the chemistry of functional sites of wool yarns. Hence, MW radiation has greatly benefited the yarns by enhancing their sorption behavior [24].

### 3.3. Mordanting

Mordants are crucial for natural dyeing of the textiles using plant-based colorants as they play a pivotal role in the enhanced dye fixation and colorfastness [27,28]. Traditionally, various salts such as Cu, Cr, Co, Ni, Mn, etc., have been used as a mordant; however, they are carcinogenic in nature, and their effluents can harm aquatic ecosystems and soil fertility [29].

Contrarily, the salts of Al/Fe and tannic acid are considered eco-friendly and are the most suited mordants for the sustainable dyeing of textiles. Now, plant-based functional biomolecules, particularly tannin-based ones, are possible alternatives. In this study, before, during, and after dyeing of wool yarns, eco-friendly additives have been employed at optimized processing conditions (Table 3) to analyze their impact on dye uptake and colorfastness.

It is obvious from the results summarized in Table 3 that both mordant type and mordanting method directly influence the adsorption of the natural dye extracted from the grape leaf extract on the wool yarns. Among the metal salts, iron sulphate (Fe^2+^) exhibited a greater enhancement in color strength (K/S = 4.01) compared to aluminum acetate (Al^3+^, K/S = 2.7166), indicating stronger dye–fiber interaction. In contrast, bio-mordants showed a more pronounced effect on dye uptake than metal salts. Among them, myrobalan resulted in the highest color strength (K/S = 5.2566), followed by red sumac (K/S = 4.310), while tannic acid (K/S = 3.4179) showed the lowest improvement among bio-mordants, yet still higher than that achieved with metal salts. These findings of our work indicate that the plant-based tannic-rich bio-mordants have the potential to be used for promoting dye adsorption during the textile dyeing techniques.

Furthermore, the shade properties of the prepared dyed yarns were described by the CIELAB color parameters, and the results are summarized in Table 3. It is obvious from the lightness (L*) values that relatively darker shades (L* = 61.92) were obtained for the Fe^2+^-treated samples, while Al^3+^ post-mordanted yarns exhibited brighter tones (L* = 74.91). It can also be seen from the results that the positive a* values (e.g., 1.11 for Fe^2+^ and 0.82 for Al^3+^) indicate only a slight reddish contribution, whereas the higher positive b* values (15.95 for Fe^2+^ and 23.72 for Al^3+^) indicate that yellow is the dominant hue imparted by the natural flavonoid dye extracted from the grape leaves. The color saturation of the prepared samples is demonstrated by the chroma (C*ab) values. The C*ab results indicated that the Al^3+^-treated samples showed higher chroma (C*ab = 23.73) compared to Fe^2+^ (C*ab = 15.99), showing more vivid coloration. Likewise, red sumac and myrobalan yielded dark shades with enhanced saturation and color depth, especially myrobalan that resulted in the highest K/S value of 5.2566. Furthermore, the hab (°) values such as 86° for Fe^2+^ and 88.02° for Al^3+^ confirm the yellowish tonal region with slight variations depending on the mordant type and method.

### 3.4. Colorfastness

Fastness properties of any dyed textiles are the first question for consumers, particularly when using natural dyes [30]. Chemical mordants form coordinate bonds with –C=O and –NH of wool and –OH of dye to shed a firm and stable shade. Depending upon the reduction power, fabric treatment, and colorant nature, shade strength is achieved. Iron sulfate interacts with dye molecules and yarn amide linkage by utilization of d-orbital, due to which its shade appearance is changed, but also its strength is enhanced. Similarly, bio-mordants act as a linkage between dye molecules and wool yarns through H-bonding, where additional conjugates present in bio-mordants play their role to furnish a good-quality shade of excellent strength. Myrobalan-based tannin molecules have utilized their –OH as a binding site with the –OH of dye (grape colorant) and –C=O and –NH of wool fabric through H-bonding and ionic interaction to develop a shade of excellent strength. During the dyeing process (pH 5), some amino functional groups of wool fibers remain positively charged, resulting in the electrostatic interaction with the negatively charged phenolic groups of the dye molecules. Conclusively, functional groups of tannins act as a bridge and facilitate the improvement of dye fixation and color strength of the resultant dyed wool.

The extract of grape leaf has a flavonoid-based yellow colorant, which has good potential to impact film color onto wool yarns after mordanting, before, and during dyeing. The metal formed a stable dye complex with yarns under selected dyeing conditions. The dyed yarns were exposed to crocking, light, and soaping processes and showed maximum resistance to fading. Similarly, organic molecules, i.e., –OH from tannic acid and –OH of tannins from myrobalan and red sumac, have formed extra H-bonding with –OH of dye and the amino part of wool yarns that have yielded colorfast shades. Hence, conjugations by biomolecules and surface tuning of yarns by MW treatment have both played their role in developing colorfast shades. The fastness rating given in Table 4 reveals that wool yarns mordanted during, before, and after dyeing with grape leaf extract have given better light (5), washing (4/5-5), and crocking (4/5-5) properties. Hence, using grape leaf-based natural colorant (flavonoid) has shown promising potential as an alternative to toxic synthetic dyes for wool yarns.

## 4. Conclusions

The new sources of plant-based dyes are always under observation because the global community is now becoming aware of toxic dyes and chemicals and their formulated products. New methods are being used to valorize their coloring behavior towards textiles through cost-effective, energy, and time-effective methods. The Box–Behnken design was used in the statistical modelling for product development and MW treatment was used for enhancing isolation yield, as well as for mordanting for stable shading; these practices have revolutionized the natural dyeing process. In this study, the dyeing of yarns with grape leaf extract of pH 5 at 80 °C for 25 min of 3 g/100 mL of salt, followed by MW treatment up to 4 min, provided excellent shade strength. Eco-friendly mordants have developed colorfast shades of woolen yarns. A statistical approach using the Box–Behnken design with radiation treatment should be used to optimize the use of dye-yielding plants for natural and synthetic yarns, and fabrics.

## Figures and Tables

**Table 1:** Response in terms of Results (K/S) for selection of dyeing variables using Box–Behnken design.

pH	Time (min)	Temperature (°C)	Salt %	K/S	Shade
3	25	70	3	2.1276	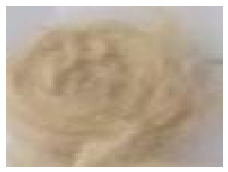
7	25	70	3	2.0452	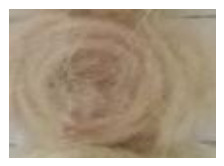
3	65	70	3	2.8966	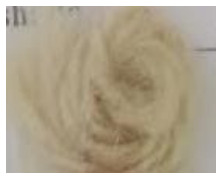
7	65	70	3	1.7097	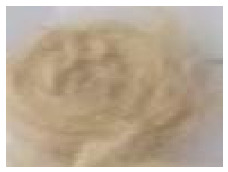
5	45	60	1	3.0733	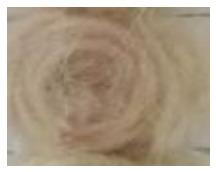
5	45	80	1	1.7315	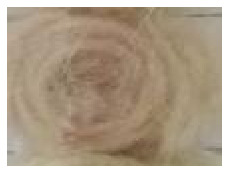
5	45	60	5	4.4744	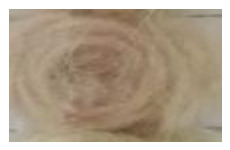
5	45	80	5	4.0939	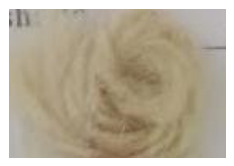
3	45	70	1	1.8688	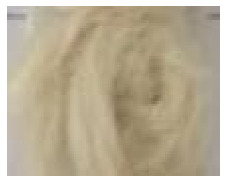
7	45	70	1	2.4381	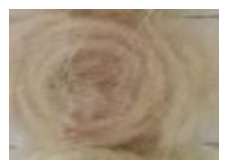
3	45	70	5	1.4393	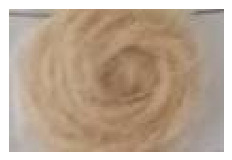
7	45	70	5	2.2698	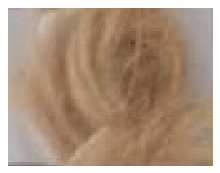
5	25	60	3	2.4720	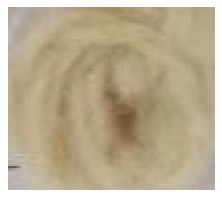
5	65	60	3	1.7444	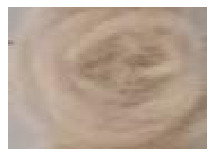
5	25	80	3	4.7719	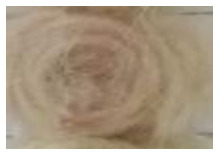
5	65	80	3	5.3088	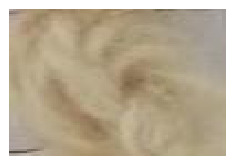
3	45	60	3	0.9587	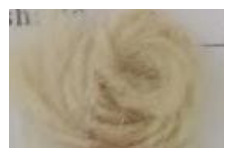
7	45	60	3	1.1879	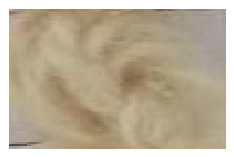
3	45	80	3	1.4973	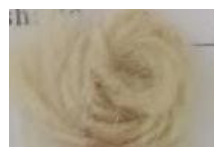
7	45	80	3	3.3169	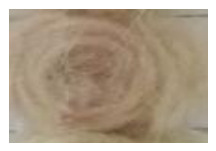
5	25	70	1	4.6406	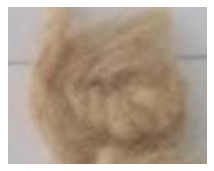
5	65	70	1	4.0452	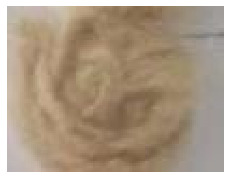
5	25	70	5	3.0348	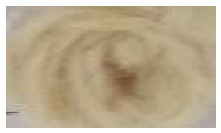
5	65	70	5	1.7968	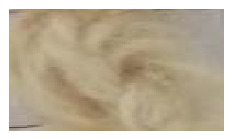
5	45	70	3	2.7086	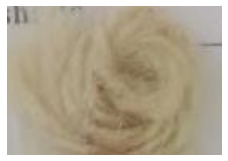
5	45	70	3	1.8171	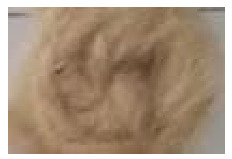
5	45	70	3	2.4343	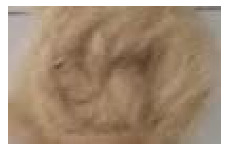

**Table 2:** Two-way analysis of variance for process optimization.

Source	DF	Adj SS	Adj MS	F-Value	*p*-Value
Model	14	23.6132	1.68666	7.18	0.003
Linear	4	11.5137	2.87843	12.25	0.001
pH	1	1.7878	1.78784	7.61	0.022
Time	1	2.6849	2.68485	11.43	0.008
Temperature	1	5.5091	5.50909	23.44	0.001
Salt	1	2.4648	2.46479	10.49	0.010
Square	4	7.8730	1.96824	8.38	0.004
pH*pH	1	0.5195	0.51948	2.21	0.171
Time*Time	1	4.1924	4.19237	17.84	0.002
Temperature*Temperature	1	0.3766	0.37661	1.60	0.237
Salt*Salt	1	0.0046	0.00456	0.02	0.892
2-Way Interaction	6	8.9062	1.48437	6.32	0.008
pH*Time	1	2.4794	2.47939	10.55	0.010
pH*Temperature	1	0.6323	0.63234	2.69	0.135
pH*Salt	1	0.0171	0.01706	0.07	0.794
Time*Temperature	1	0.1737	0.17368	0.74	0.412
Time*Salt	1	0.1032	0.10323	0.44	0.524
Temperature*Salt	1	6.0027	6.00269	25.55	0.001
Error	9	2.1149	0.23498		
Lack-of-Fit	7	1.6979	0.24255	1.16	0.536
Pure Error	2	0.4170	0.20849	-----	-----
Total	23	25.7281			
**Model Summary**	**S**	**R-sq**	**R-sq (adj)**	**R-sq (pred)**	-----
	0.484752	91.78%	78.99%	42.94%	-----

**Table 3:** Color depth and shade value of dyed wool yarns before, after, and during mordanting.

Mordanting Agent	Mordanting Method	K/S	L*	a*	b*	C*	h	Shades
Iron sulphate	Pre	4.0194	61.92	1.11	15.95	15.99	86.00	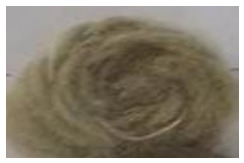
	Post	2.7116	62.76	−0.73	9.12	9.15	94.56	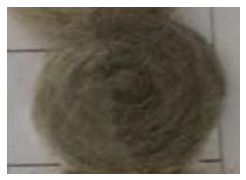
	Meta	3.1444	62.75	−0.64	11.25	11.27	93.27	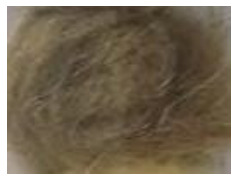
Aluminum acetate	Pre	2.0882	77.37	−0.41	22.58	22.59	91.04	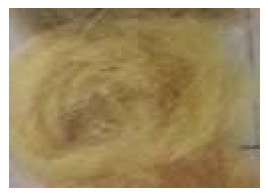
	Post	2.7186	74.91	0.82	23.72	23.73	88.02	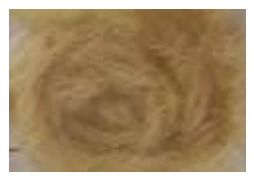
	Meta	2.2722	75.61	0.21	22.07	22.07	89.47	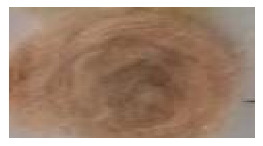
Tannic acid	Pre	1.8567	72.22	3.82	13.32	13.85	74.00	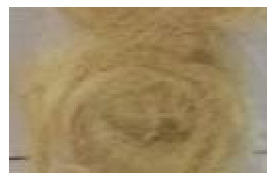
	Post	3.3183	68.87	4.71	16.56	17.21	74.11	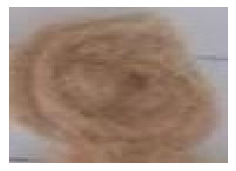
	Meta	3.4179	70.58	4.70	19.35	19.91	76.33	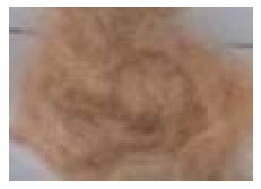
Red sumac	Pre	4.1301	59.27	2.90	11.48	11.84	75.83	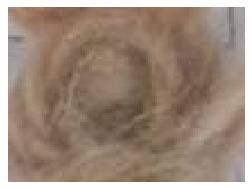
	Post	3.4122	62.60	4.33	10.51	11.37	67.63	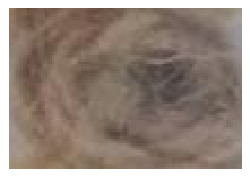
	Meta	4.3100	62.66	3.59	13.69	14.16	75.29	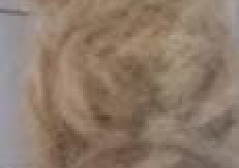
Myrobalan	Pre	5.2566	69.56	2.22	23.58	23.68	84.63	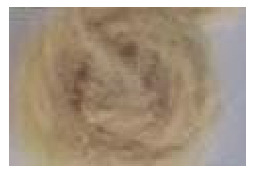
	Post	4.8448	69.23	2.73	22.95	23.12	83.21	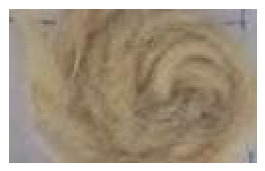
	Meta	4.2900	70.60	2.26	21.68	21.80	84.06	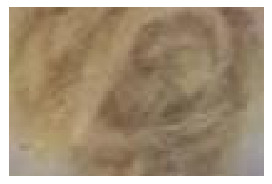

**Table 4:** Colorfastness properties of mordanted wool yarns dyed using grape leaves extract.

Mordanting Agent	Mordanting Method	Light Fastness	Washing Fastness	Dry Rubbing Fastness	Wet Rubbing Fastness
Iron sulphate	Pre	5	4/5	5	5
	Post	5	4/5	5	4/5
	Meta	5	4	5	4/5
Aluminum acetate	Pre	5	4/5	5	4/5
	Post	5	4/5	5	5
	Meta	5	4/5	5	4/5
Tannic acid	Pre	5	4/5	5	4/5
	Post	5	4/5	5	4/5
	Meta	5	4/5	5	5
Red sumac	Pre	5	4/5	5	4/5
	Post	5	4/5	5	4/5
	Meta	5	4/5	5	5
Myrobalan	Pre	5	4/5	5	5
	Post	5	4/5	5	4/5
	Meta	5	4/5	5	4/5

## Data Availability

Data is contained within the article. Dataset is available from the authors on request.
